# Ischemic Stroke in a Patient With Atrial Tachycardia, Methylenetetrahydrofolate Reductase Mutation and New-Onset Atrial Fibrillation: Is Early Initiation of Anticoagulation Therapy Indicated?

**DOI:** 10.7759/cureus.9420

**Published:** 2020-07-27

**Authors:** Harry O Eyituoyo, Nkechi C Arinze, Rieta N Aben, Felix Sogade

**Affiliations:** 1 Internal Medicine/Community Medicine, Mercer University School of Medicine, Macon, USA; 2 Electrophysiology, Georgia Arrhythmia Consultants and Research Institute, Macon, USA

**Keywords:** atrial tachycardia, atrial fibrillation, methylenetetrahydrofolate reductase mutation, ischemic stroke, anticoagulant therapy, loop recorder, thrombolytics, hypercoagulable, homocysteinemia, chadsvasc score

## Abstract

Atrial fibrillation is the most common dysrhythmia, affecting about 6 million people in the United States. Atrial fibrillation has been shown to be an independent risk factor for stroke. Atrial tachycardia are common findings on Holter monitoring in the general population and may be associated with the development of atrial remodeling and atrial fibrillation inducibility. Studies have shown that atrial tachycardia is associated with the development of atrial fibrillation and subsequent stroke. The American Heart Association current guidelines recommend the use of oral anticoagulants in patients with atrial fibrillation and an elevated CHA2DS2-VASc ≥2 in men or ≥3 in women. However, anticoagulant therapy is not currently recommended in patients with atrial tachycardia despite increasing evidence of its association with the development of stroke.

We report the case of a 68-year-old woman with a past medical history significant for repetitive atrial tachycardia and methylenetetrahydrofolate reductase mutation who presented to an outside emergency department following a fall, weakness and associated aphasia while in atrial fibrillation. Thrombolysis and control of the patient’s rhythm resulted in an initial improvement in the patient symptoms and reversal to normal sinus rhythm. She was subsequently referred to a tertiary stroke center for further management.

## Introduction

Atrial fibrillation (AF) is the most common dysrhythmia, affecting about 6 million people in the United States [1]. AF is a quivering and irregular heart beat characterized on an electrocardiogram by the loss of P-waves with duration greater than 30 seconds, absence of an iso-electric baseline and variable ventricular rate. Ineffective atrial emptying in AF promotes clot formation, which leads to an increased risk of stroke. AF has been shown to be an independent risk factor for stroke [2]. Atrial tachycardia (AT) are common findings on Holter monitoring in the general population, which may be associated with the development of atrial remodeling and AF inducibility [3]. Studies have shown that AT is associated with the development of AF and subsequent stroke [4-5]. The American Heart Association (AHA) current guidelines recommend the use of oral anticoagulants in patients with AF and an elevated CHA2DS2-VASc score of ≥2 in men or ≥3 in women [6]. However, anticoagulant therapy is not currently recommended in patients with AT despite increasing evidence of its association with the development of stroke.

We report a case of two episodes of ischemic stroke in a patient with short repetitive AT, new-onset AF and methylenetetrahydrofolate reductase mutation (MTHFR).

## Case presentation

**Figure 1:**
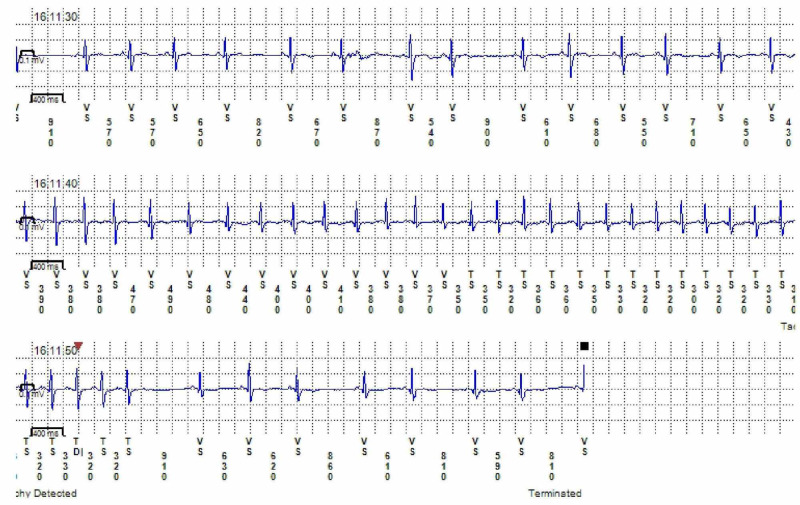
Loop recording showing atrial tachycardia.

A 68-year-old, Caucasian female with a past medical history of asthma, scleroderma and MTHFR mutation (Variant: C677T) has been followed up in our electrophysiology clinic for AT and frequent premature ventricular complexes. She had a loop recorder implant three years ago. The loop recorder was placed due to a transient ischemic attack (TIA) with intraocular changes identified by her ophthalmologist. Her loop recorder has consistently shown an average of 2-3 short runs of repetitive AT for 4-6 beats per day (Figure 1), but never documented AF. She presented to an outside emergency department (ED) following a fall, aphasia and weakness after her spouse found her on the floor. Initial National Institute of Health Stroke Score (NIHSS) was 2 (1 point for loss of consciousness and 1 point for aphasia), blood pressure (BP) was 170/97 mmHg, random blood glucose was 103 mg/dl. Electrocardiography (EKG) showed the patient to be in AF with rapid ventricular response (RVR) with a heart rate (HR) of 219 beats per min (bpm). Troponins were unremarkable. She was given alteplase with improvement of symptoms for presumed left middle cerebral artery (MCA) embolic stroke and diltiazem for AF.

The patient was then transferred to a tertiary care stroke center for further management. On arrival at the tertiary center, the patient’s NIHSS was 3 (increased from 2), she was in normal sinus rhythm (NSR) and diltiazem was discontinued. Physical examination revealed an HR of 68 bpm; BP was 119/53 mmHg. On neurological examination, she could not remember the year (but remembers the month and day); naming, repetition and comprehension were impaired. On motor examination, there was minimal decrease in the right hand-grip and right triceps. There was right lower extremity-drift and a decreased motor strength of 4/5. There was full motor strength of 5/5 on the left upper and lower extremity, sensory examination was intact throughout, and deep tendon reflexes were 2+. The patient was admitted to the neuro intensive care unit (NICU) for further stroke workup. Transthoracic echocardiography showed an ejection fraction (EF) of 65-70%, with small circumferential pericardial effusion. Magnetic resonance imaging (MRI) showed a small patchy area of diffusion restriction involving the left temporoparietal region. Magnetic resonance angiography (MRA) also showed short segment stenotic lesions of the proximal left inferior M2 division. Carotid ultrasound was negative for any thrombus; there was a plaque in both bifurcations of no hemodynamic significance. The patient was started on metoprolol, aspirin, and atorvastatin. The electrophysiology team was consulted to evaluate the loop recorder.

She continued to have expressive aphasia with irregularly irregular HR patterns. The patient continued falling in and out of AF with palpitations, HR increased to 160-180s. EKG revealed AF with ventricular rates into the 150s, non-specific ST segment and T wave changes, with no evidence of acute ischemia. Telemetry showed variable flutter and AF rates in the 150s-180s. Metoprolol was withheld on the second day of hospital admission due to bradycardia. Amiodarone and apixaban (CHA2DS2VASC was 4 for age > 65, gender and stroke) were commenced.

On the fourth day of hospital admission, the patient had yet another stroke evidenced by dysarthria, left-sided weakness with right gaze deviation. NIHSS was 17. CT head w/o contrast showed an acute thrombus of right MCA at the mid M1 segment with no distal filling past defect; CT angiography neck w/o contrast was unremarkable. Alteplase could not be given as the patient had been started on apixaban overnight. The patient underwent right MCA thrombectomy with TICI3 flow through the right MCA after the first pass. CT head w/o contrast post procedure was negative for any hyperdensity or early infarct changes. She continued aspirin; apixaban was discontinued. While in the NICU, the patient was noted to have asymptomatic bradycardia in the 40s and hypotension while sleeping. She was treated with IV norepinephrine and oral midodrine; with careful fluid resuscitation and gentle diuresis her BP and HR normalized. She went into AF with RVR and spontaneously reverted to NSR. She remained hemodynamically stable with BP in 100-140s and normal HR with irregularly irregular patterns. The patient had marked recovery of function post-intervention for her second stroke with recommendations from physical therapist for outpatient rehabilitation. Apixaban was resumed. She was discharged on hospital day 7 in a stable condition, with electrophysiology and neurology follow-up. At the neurology clinic follow-up two months later, the patient reported mild language difficulties, but she was markedly improved.

## Discussion

There is growing literature on the association between AT and ischemic stroke, and the need of CHA2DS2VASC score for risk stratification for this finding [7-10]. The pathophysiology of cardio-embolic stroke is not fully understood but studies have linked hypercoagulability with an increased risk of stroke. Larsen et al. observed that in the middle-aged and older populations, excessive supraventricular ectopic activity was associated with an increased risk of ischemic stroke beyond incident AF. Stroke was more often the first clinical presentation, rather than AF, in these study subjects [5]. As per AHA guidelines, there is no consensus on the use of anticoagulation in AT or in AT with predisposing thrombotic factors [6].

Studies have shown an association between AF and stroke [2, 11]. Current AF management guidelines require the use of oral anticoagulants in patients with CHA2DS2-VASc score of ≥2 in men or, ≥3 in women to reduce the risk of stroke. Possible explanations for stroke risk in AF include factors that activate the Virchow triad such as stasis in the left atrium (LA) and left atrial appendage (LAA), endothelial damage from atrial structural remodeling and induction of a prothrombotic and hypercoagulable state. These mechanisms lead to enhanced platelet activation and initiation of the coagulation cascade, which causes the formation of intracardiac and extra cardiac thrombus leading to embolism.

Several studies have shown that increased levels of homocysteine are a risk factor for vascular disease like myocardial infarction and stroke [12, 13]. MTHFR (Variant: C677T) is a genetic mutation on the enzyme that breaks down the amino acid homocysteine level in the blood. The mutation affects the enzyme ability to function properly or inactivate it. This can cause homocysteinemia, a hypercoagulable state with increased risk of thrombosis [14]. There is no recommendation on the use of antithrombotic therapy as a primary prevention of stroke in patients with hyperhomocysteinemia. There is paucity of data on the incidence of ischemic stroke in patients with both MTHFR and AF, although both independently increase the risk of stroke [11, 13].

In our case, the loop recorder was placed due to a TIA with intraocular changes identified by her ophthalmologist. The recorder consistently picked up short runs of repetitive AT without having clinical AF. The first activation of AF coincided with the occurrence of her first ischemic event in the ED. The AF duration was approximately two hours before initial antithrombotic therapy was administered. She later had another episode of ischemic stroke while in the hospital. Our patient had multiple risk factors that increased her stroke risk, including history of AT, MTHFR (Variant: C677T) and scleroderma. These compounding factors have been linked to an increased risk of thrombosis and possible stroke. These factors raise a concern as to who gets an anticoagulant therapy. There is a need to stratify patients with AT using a scale similar to CHA2DS2VASC score and determine at what point it is deemed safest to initiate treatment in such patients to prevent incident stroke. It remains to be answered if early anticoagulation therapy would have prevented or reduced the risk of the two ischemic strokes in this patient.

To our knowledge, this is the first reported case of two ischemic strokes and incident AF in a patient with short repetitive AT and MTHFR (Variant: C677T). Although causality cannot be inferred from our case report, it is imperative for future studies to be conducted on these patients to ascertain the risk of ischemic stroke and the initiation of anticoagulation therapy in AT.

## Conclusions

Anticoagulant therapy might be important in patients with short repetitive AT. Additional consideration beyond CHA2DS2VASc score should be made for such patients with high thrombogenic risk factors. Further studies are warranted to assess the significance of anticoagulant therapy in these patients.
